# Vγ9Vδ2 T cells strengthen cisplatin inhibition activity against breast cancer MDA-MB-231 cells by disrupting mitochondrial function and cell ultrastructure

**DOI:** 10.1186/s12935-021-01815-0

**Published:** 2021-02-16

**Authors:** Xin Huang, Cunchuan Wang, Ningxia Wang

**Affiliations:** Department of Breast Surgery, The First Affiliated Hospital, Jinan University, 613 West Huangpu Road, Guangzhou, 510630 Guangdong People’s Republic of China

**Keywords:** Vγ9Vδ2 T cell, Cisplatin, MDA-MB-231 cells, Inhibitory effect

## Abstract

**Background:**

Breast cancer ranks second of new cases and fifth of death in 2018 worldwide. Cis-platinum (CDDP) has been used as a chemotherapy to treat breast cancer for years. However, CDDP can adversely disrupt immune function of host. Thus, development of new protocol that can minimize side effect and meanwhile elevate clinical efficacy of CDDP will eventually benefit cancer patients. Since Vγ9Vδ2 T cells can up-regulate immune function of cancer patients, therefore, our hypothesis is that introduction of Vγ9Vδ2 T cells could potentiate CDDP efficacy against breast cancer.

**Methods:**

We used breast cancer cell line MDA-MB-231 as model cell to test our hypothesis. The cancer cell viability in vitro in the context of different dose of CDDP was analyzed by flow cytometry. The cytoskeleton alteration was visualized by confocal microscopy, and the ultrastructure of cell membrane was observed by atomic force microscopy. The mitochondrial function of MDA-MB-231 cells was detected as well by flow cytometry.

**Results:**

Comparing to either Vγ9Vδ2 T cells or CDDP alone, Vγ9Vδ2 T cells plus CDDP could more strikingly induce MDA-MB-231 cell membrane ultrastructure disruption and cytoskeleton disorder, and more significantly enhance the inhibition of CDDP on proliferation of MDA-MB-231 cells. At the same time, Vγ9Vδ2 T cells strengthened CDDP-induced mitochondrial dysfunction of cancer cells.

**Conclusion:**

This work revealed that Vγ9Vδ2 T cells could synergistically enhance the inhibition activity of CDDP against breast cancer cells. Meanwhile, this in vitro proof-of-concept study implied the clinical prospect of the combining application of Vγ9Vδ2 T cells and CDDP in breast cancer therapy.

## Background

Breast cancer is one of leading causes of cancer death in women worldwide. Currently, clinical treatments against breast cancer mainly include surgery, chemotherapy, radiotherapy, endocrine and molecularly targeted therapy. Among these protocols, chemotherapy is routinely used to treat breast cancer, although the severe side effects have raised a lot of concerns. For instance, chemotherapy drugs can generate pro-tumorigenic and pro-metastatic effects [1], and promote cancer cell evolution, which contribute to cancer recurrence and resistance to anti-tumor therapy [2]. Currently, increasingly scientific evidences revealed the evil part of chemotherapy drugs in tumor treatment, including promotions of metastasis, proliferation, immune escape, and so on [1–3]. Therefore, to develop new treatment strategies for cancer based on biomarkers has been under continuous investigation during the past a few year [4–14]. One of the most highlighted progresses is the successful achievement of chimeric antigen receptor (CAR) T cells in B cell lymphoma [10–13, 15]. This set a new paradigm for cancer treatment using immune cells.

Vγ9Vδ2 T cell belongs to one subset of human peripheral γδ T cell, one major component of T lymphocytes (the other is αβ T cell). Vγ9Vδ2 T cells showed promising clinical value because of the potent anti-tumor activity [16–18], thus could be developed into a new strategy [13, 16, 17, 19–21] for breast cancer immunotherapy. For example, Vγ9Vδ2 T cells could inhibit breast cancer cell proliferation by regulating crucial molecules related to cell survival and apoptosis [22]; Vγ9Vδ2 T cells exerted promising breast tumor inhibition activity in the context of zoledronic acid pre-treatment [23]; notably, clinical Phase I trial study on breast cancer patients revealed that sustained population of Vγ9Vδ2 T cells were positively correlated with sound prognosis [24]. Therefore, previous reports altogether indicated that Vγ9Vδ2 T cells-based immunotherapy will be one of promising therapeutic approach for breast cancer [13, 20, 25].

In this work, we proposed a new protocol by combining chemotherapy drug cis-platinum (CDDP) and Vγ9Vδ2 T cell to treat a selected breast cancer model cell line MDA-MB-231, and tried to reveal the in vitro efficacy of this combination from both large-scale number of cells as well as single cell level. We used flow cytometry, atomic force microscopy and confocal microscopy to examine mitochondrial function, cell ultrastructure and cytoskeletal organization. We found that, comparing with single treatment alone, CDDP plus Vγ9Vδ2 T cells exhibited significant greater inhibition against MDA-MB-231 cell growth, elevated mitochondrial dysfunction, ultrastructural and cytoskeletal impairments, implicated with Vγ9Vδ2 T cells could potentiate CDDP inhibition activity against breast cancer cell MDA-MB-231. This proof-of-concept work provided a preliminary clue for development of new clinical treatment protocol (e.g. immune cells plus chemotherapy drugs) for breast cancer, eventually benefiting patients with breast cancer.

## Materials and methods

### γδ T cell isolation and culture

The methodology of γδ T cell isolation and culture had been described in detail in previous reports [16, 17, 26, 27]. Briefly, the peripheral blood was collected from healthy donors, then peripheral blood monocyte cells (PBMCs) were isolated using the Ficoll-Paque centrifugation protocol, following by culturing PBMCs in RPMI-1640 culture medium supplemented with 10% fetal bovine serum (FBS), IL-2 (5 ng/ml) and zoledronate (10 nM). PBMCs were seeded in 24 or 48 well-plates. This culture medium recipe could selectively expand Vγ9Vδ2 T cells from PBMCs. After cultured for 10–12 days, the expanded Vγ9Vδ2 T cells were used to conduct experiments. The purity of Vγ9Vδ2 T cells was assayed using flow cytometry, and only those exceeded 90% of purity were used in our following experiments.

### MDA-MB-231 cell viability assay

Breast cancer cell line MDA-MB-231 (MDA-MB-231, Tam1, ATCC® CRL-3435™) was used as a cell model in our work. Cells were cultured in RPMI-1640 culture medium containing 10% BSA, and cells were used for experiments after the confluency reached 70–80%. For cis-platinum (CDDP) treatment, three CDDP dosages include 120 μM, 240 μM, and 480 μM were used, and treatment times of 24 h and 48 h were applied. For T cell treatment, Vγ9Vδ2 T cell number was determined according to effector (Vγ9Vδ2 T): target (MDA-MB-231) ratios (1:1, 5:1, and 10:1), the 6 h of cell–cell interaction was applied here. As for combined treatments, MDA-MB-231 cells were firstly treated with CDDP (240 μM) for 24 h, followed T cell incubation (5:1) for 6 h after cells were twice washed to remove extra CDDP. Then MDA-MB-231 cells were collected and stained with Propidium Iodide (PI) for 5 min at room temperature, and flow cytometry was used to analyze the dead cell percentage.

### Mitochondrial function assay of MDA-MB-231 cells

To analyze mitochondrial function of MDA-MB-231 cells in the presence and absence of CDDP or Vγ9Vδ2 T cells, mitochondrial ROS detection assay kit (Sigma), MitoTracker (Thermofisher) staining and mitochondrial membrane potential assay kit (Sigma) were applied in our work, and sample preparation procedures referred to the standard protocols provided by the reagent providers. Afterward, cells were analyzed by flow cytometry. All obtained data by flow cytometry were further analyzed with FlowJo (FLOWJO, LLC) software.

### Atomic force microscope visualization of membrane ultrastructure

To visualize membrane surface nanostructures, atomic force microscopy (AFM) (Bioscope Catalyst, Bruker) was applied to scan single MDA-MB-231 cells. The detailed methodology for AFM principle and sample preparation could be detailed referred in previous published works [28–33]. In brief, the tapping mode AFM was used to scan cells at room temperature in air. The spring constant of the cantilever was calibrated at 0.06 ~ 0.11 N/m. The average roughness (*R*_a_) that describes topography properties of membrane surface was obtained according to the following formula [31]:

$$ R_{a} = \frac{1}{N}\sum\limits_{n - 1}^{N} {\left| {z_{n} - \overline{z}} \right|} $$

where N represents the total number of data points in a selected area, z n is the height of the n th point and $\bar{z}$ is the mean height.

### Cytoskeleton visualization using confocal microscopy imaging

To achieve cytoskeleton (F-actin and tubulin-α) visualization, fluorescence staining was performed by following Invitrogen standard immunofluorescence staining protocol. Staining was performed at room temperature. FITC-phalloidin was used to stain actin, and mouse anti-human fluorescent tubulin-αantibody was used to stain tubulin-α. Cell nucleus was stained with dye DAPI. Cells were washed with PBS between each step, then anti-fade reagent was added before performing confocal analyses. The confocal microscopy system used in our work was Leica SP8, and 60× oil immersed objective was applied for imaging.

### Data analyses and statistics

All statistical results were expressed as mean ± SEM (Standard Error of the Mean). To analyze statistical significance between experimental groups and control group, one-way ANOVA was applied to compare the difference between the control group and each experimental group. Statistical significance: ns, no significance; *, p < 0.05; **, P < 0.01; ***, P < 0.001; ****, P < 0.0001.

## Results

### Both cis-Platinum and Vγ9Vδ2 T cells inhibited MDA-MB-231 viability in a dose-dependent manner

**Fig. 1:**
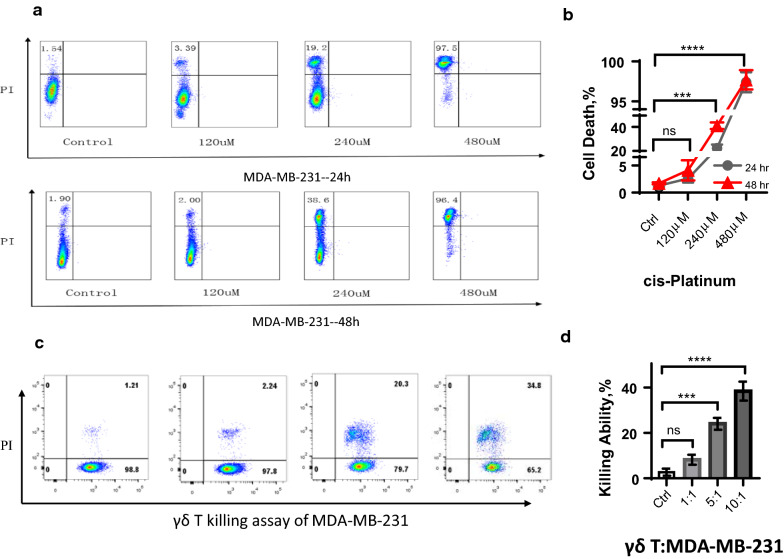
Cell viability and killing assay of MDA-MB-231 cells in the presence of cis-platinum (CDDP) and Vγ9Vδ2 T cells. **a** Representative flow cytometry graphs of cell death detection of MDA-MB-231 cells. **b** Statistical graph of MDA-MB-231 cell viability in the absence and presence of CDDP, showing CDDP induced cell death in a dose-dependent manner. **c**, **d** Killing assay results of MDA-MB-231 cells by Vγ9Vδ2 T cells. ns, no significance; ***P < 0.001; ****P < 0.0001

To determine inhibition concentration of cis-platinum and incubation time with MDA-MB-231 cells, we firstly used flow cytometry to analyze cell viability in the presence of cis-platinum (Fig. 1a). The analyzed results showed that, for 24 h of incubation, 120 μM of cis-platinum did not inhibit cell viability statistically, whereas 240 μM and 480 μM could induce cell death significantly, from 1.29 ± 0.13 (control, %) to 21.47 ± 1.79 (%) and 97.4 ± 0.72 (%), respectively. As for 48 h of incubation, the percentage of cell death increased from 1.7 ± 0.12 (control) to 41.13 ± 1.68 and 98.1 ± 0.7 after incubated with 240 μM and 480 μM of cis-platinum, respectively (Fig. 1b). Furthermore, we conducted killing assay to analyze the inhibiting efficacy of Vγ9Vδ2 T cells against MDA-MB-231 cells, and results were shown in Fig. 1c, d. It indicated that when the ratio of effector (Vγ9Vδ2 T cells): target (MDA-MB-231) reached at 5:1, the inhibition efficacy (24 ± 1.53, %) against MDA-MB-231 could be significantly higher than the control group (2.67 ± 0.9, %) (Fig. 1d) after 6 h of co-incubation. Moreover, the inhibition efficacy increased to 38.33 ± 2.4% for the E:T = 10:1 group. These results altogether indicated that both cis-platinum and γδ T cells inhibited MDA-MB-231 viability in a dose-dependent manner.

### Vγ9Vδ2 T cells could strength cis-Platinum inhibition activity against MDA-MB-231

**Fig. 2:**
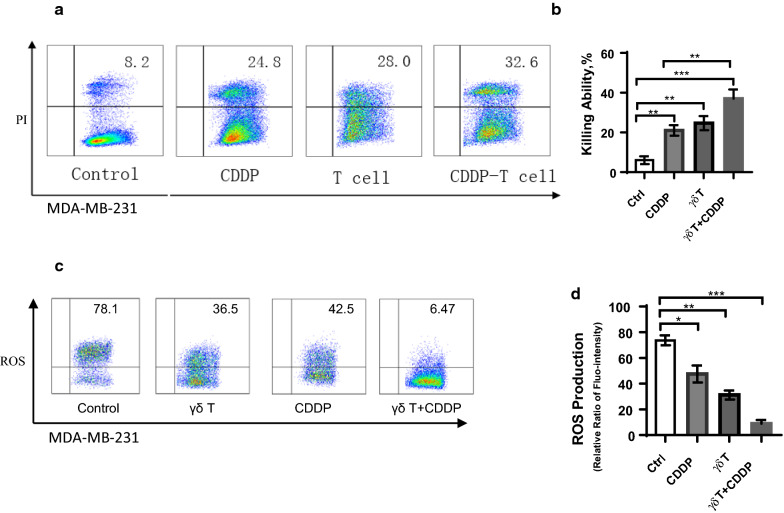
**a** Flow cytometry plot analyses of MDA-MB-231 cell viability in the context of treatment of cis-platinum (CDDP) plus Vγ9Vδ2 T cells, showing data of one time of experiment. **b** Statistical data from triple repetitions of experiments, showing that Vγ9Vδ2 T cells could significantly strengthen CDDP cytotoxicity against MDA-MB-231. **c** Flow cytometry plot data of reactive oxygen species (ROS) production from one time of experiment. **d** Triple repetitions, clearly indicating that Vγ9Vδ2 T cells could dramatically strengthen CDDP-induced reduction of ROS production of in MDA-MB-231 cells. *p < 0.05; **P < 0.01; ***P < 0.001

Furthermore, we tried to check whether the combination of cis-platinum (CDDP) and Vγ9Vδ2 T cells could exert stronger inhibiting efficacy than one alone. According to results shown in Fig. 1, since 120 μM cis-platinum failed to statistically inhibit cell viability but 480 μM cis-platinum killed > 97% cells, we thus selected 240 μM as work concentration and 24 h as incubation time for cis-platinum, and 5:1 as effector: target for γδ T cells. We found that the combination of cis-platinum and Vγ9Vδ2 T cells could significantly elevate the inhibition against MDA-MB-231 compared with the control group as well as CDDP alone (Fig. 2a). Specifically, the inhibition percentage of MDA-MB-231 for control group and CDDP was 6 ± 1.15 and 21 ± 1.53, respectively, whereas 37 ± 2.65 for combination group (Fig. 2b). Together, the present results implied that combination had significant superiority compared with CDDP alone or Vγ9Vδ2 T cells alone.

### Combination of Vγ9Vδ2 T cells and cis-Platinum dramatically suppressed ROS production

Given one of inhibition mechanism of cis-platinum (CDDP) against breast cancer cells is to destruct mitochondrial respiration and metabolism, we thus detected mitochondrial alterations of MDA-MB-231 in the context of CDDP and Vγ9Vδ2 T cell treatment. Here, we analyzed the reactive oxygen species (ROS) of MDA-MB-231 using flow cytometry (Fig. 2c). The statistics showed that ROS production significantly decreased after cells treated with either CDDP or Vγ9Vδ2 T cells, from 73.7 ± 2.22 (control) to 47.5 ± 3.82 (CDDP) and 31.17 ± 2.05, and CDDP plus Vγ9Vδ2 T cells further decreased ROS production to 8.92 ± 1.67 (Fig. 2d). Such results suggested mitochondrial function of MDA-MB-231 cells were impaired in the presence of CDDP, Vγ9Vδ2 T cells and their combination could further aggravate such impairment.

### Combination of Vγ9Vδ2 T cells and cis-Platinum induced the most significant loss of mitochondrial mass and membrane potential

**Fig. 3:**
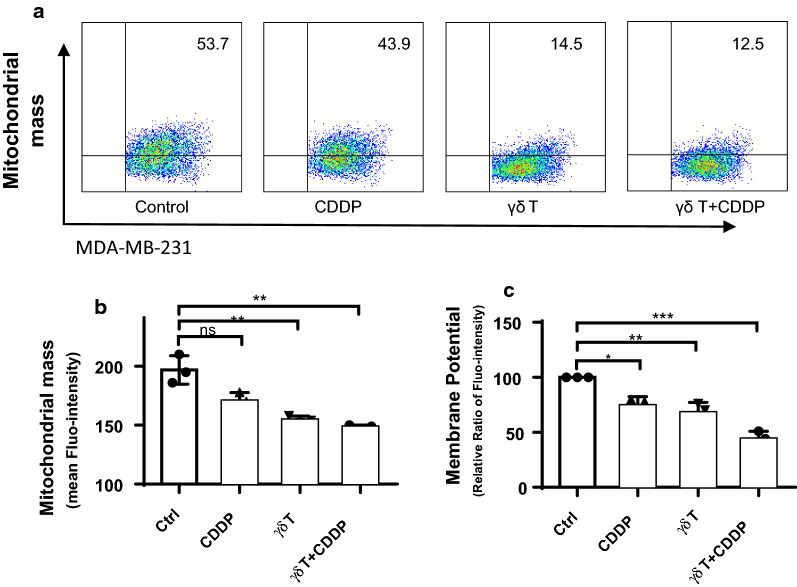
Vγ9Vδ2 T cells apparently potentiated CDDP-induced mitochondrial dysfunction including mitochondrial mass and membrane potential. **a** Representative flow cytometry graphs. **b** Statistical graph of loss of mitochondrial mass in the context of treatments. **c** Statistical graph of loss of mitochondrial membrane potential. ns, no significance; *p < 0.05; **P < 0.01; ***P < 0.001

Because both CDDP and Vγ9Vδ2 T cells could reduce ROS production in MDA-MB-231, it’s of interest to further reveal how mitochondrial function were damaged. We therefore investigated changes of mitochondrial mass and mitochondrial membrane potential using flow cytometry plus fluorescence labelling, and results were shown as Fig. 3. Figure 3a showed representative flow cytometry graphs of mitochondrial mass of fluorescence dye MitoTracker pre-labelled MDA-MB-231 cells. According to statistical analyses (Fig. 3b), we could easily see that both γδ T cells and CDDP plus Vγ9Vδ2 T cells significantly reduced mitochondrial mass in MDA-MB-231 cells by ~ 25%. Furthermore, we analyzed membrane potential of mitochondria in MDA-MB-231, which is one of the most important indicators of mitochondrial function. We discovered that the membrane potential was dramatically suppressed to approximate 75% of the control in the presence of CDDP or γδ T cells, and combination of CDDP and γδ T cells decreased it to ~ 50% of the control group (Fig. 3c). These results altogether indicated mitochondrial function was inhibited in the presence of CDDP, Vγ9Vδ2 T cells, and CDDP plus Vγ9Vδ2 T cells induced the most significant loss of mitochondrial mass and membrane potential in MDA-MB-231 cells.

### Vγ9Vδ2 T cells potentiated destruction of MDA-MB-231 membrane nanostructures induced by cis-Platinum

**Fig. 4:**
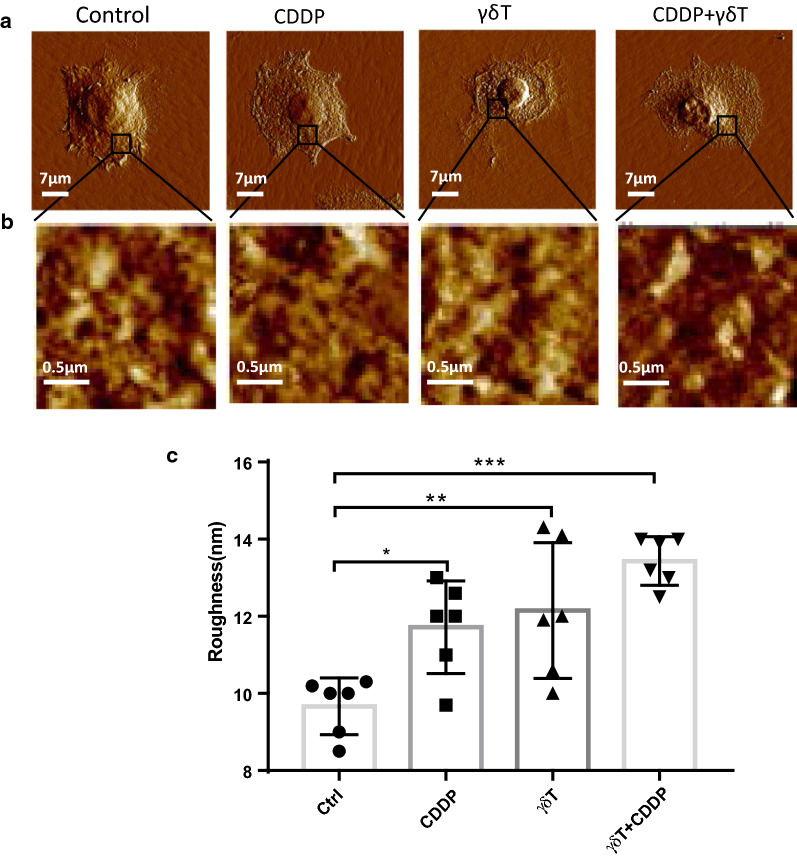
Atomic force microscopy (AFM) visualization of membrane surface ultrastructure of MDA-MB-231 cells. **a** Representative topography images of single cells, scanning size: 70 μm. **b** Membrane surface nanostructure images zoomed in from corresponding images in (A), scanning size: 2 μm. **c** Statistical comparison of surface average roughness. *p < 0.05; **P < 0.01; ***P < 0.001

To exert inhibition effects to MDA-MB-231 growth, cis-platinum (CDDP) uptake by MDA-MB-231 cells or cell–cell interaction between Vγ9Vδ2 T and MDA-MB-231 cells is required. Therefore, the earliest alteration in MDA-MB-231 might occurred on cell membrane. This made it of importance to detect ultrastructural changes in the context of treatments. We thus used atomic force microscopy (AFM) to visualize and analyze the potential alterations of MDA-MB-231 cell membrane, and results are shown in Fig. 4. Figure 4a showed topography images of single MDA-MB-231 cells, no obvious changes could be identified from these images. We further scanned membrane surface ultrastructure, and found that membrane integrity tended to be impaired, particularly for cells treated with CDDP plus Vγ9Vδ2 T cells (Fig. 4b). Since membrane surface average roughness is one of optimal parameters for describing membrane surface topography, it was statistically analyzed (Fig. 4c). We found that membrane surface roughness was significantly elevated after cells treated with both CDDP and Vγ9Vδ2 T cells. For CDDP plus Vγ9Vδ2 T cell treated group, the roughness was elevated to 13.43 ± 0.26 nm from 9.67 ± 0.3 nm of the control group (Fig. 4c). Therefore, AFM visualization and analyses indicated that Vγ9Vδ2 T cells could further potentiate destruction of MDA-MB-231 membrane nanostructures induced by cis-Platinum.

### Combination of Vγ9Vδ2 T cells and cis-Platinum destructed MDA-MB-231 cytoskeleton the most significantly

**Fig. 5:**
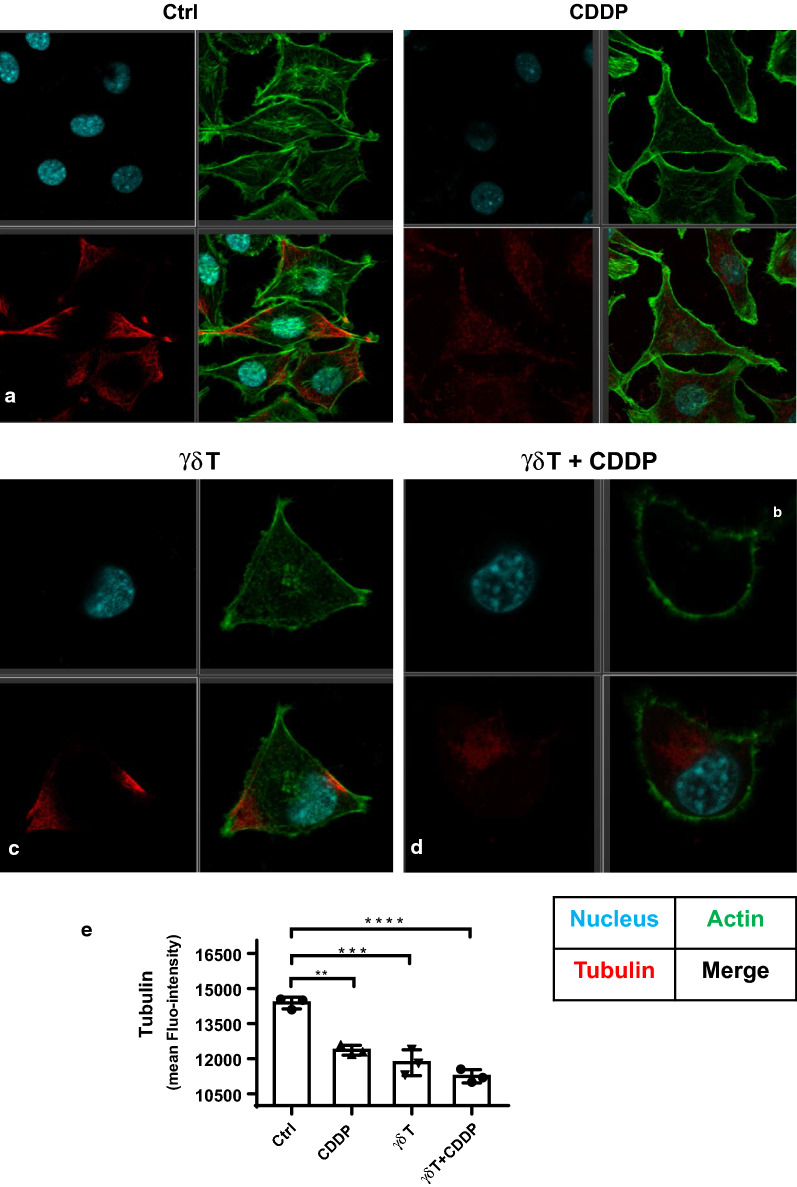
Confocal microscopy visualization of cytoskeleton. **a**–**d** Cytoskeletal changes in the absence and presence of CDDP, Vγ9Vδ2 T cells and CDDP plus Vγ9Vδ2 T cells were visually detected by Leica SP8 laser scanning confocal microscopy. **e** Quantification analyses of tubulin content of MDA-MB-231 cells in the context of treatments by flow cytometry. **P < 0.01; ***P < 0.001; ****P < 0.0001

Since cytoskeleton plays crucial roles in the multiple biological processes, including cell shape, migration, motility, mitosis, cell–cell communication, and signal transduction and so on, it’s necessary to evaluate how the cytoskeleton structures of MDA-MB-231 was impacted in the context of CDDP or Vγ9Vδ2 T cell treatment. Here, we applied confocal microscopy and flow cytometry to visualize and quantify cytoskeletal alterations, as shown in Fig. 5. For untreated cells, we could clearly see the actin (green fluorescence), one of main cytoskeletal components, appeared to be filamentous and well-organized; as for tubulin, another main cytoskeletal component, mainly localized at polar sites of cells (Fig. 5a). However, after MDA-MB-231 cells were treated with CDDP (Fig. 5b) or Vγ9Vδ2 T cells (Fig. 5c), the cytoskeletal structures were destructed, the fluorescence became dimmed and the filamentous structures could not be distinguished clearly. As for combination of CDDP and Vγ9Vδ2 T cells, both cytoskeletal components actin and tubulin were completely damaged, as evidenced by loss of fluorescence signal (Fig. 5d). Moreover, flow cytometry was also used to confirm confocal results, as it can provide statistical analyses based on large number of cells. From the flow cytometry results (Fig. 5e), we could clearly tell that both CDDP and Vγ9Vδ2 T cells could destruct the cytoskeletal organization of MDA-MB-231 cells, and combination of Vγ9Vδ2 T cells and cis-Platinum destructed MDA-MB-231 cytoskeleton the most significantly.

## Discussion

Cis-platinum has been used as clinical therapy drug against breast cancer for many years, however, its severe side effects limited the treatment efficacy clinically. Vγ9Vδ2 T cell has been increasingly recognized as new therapy strategy for cancer, since this cell could regulate immune function of the patients [16, 17, 20]. Immunotherapy including immune cells-based approach has become a new frontier of tumor therapy, but chemotherapy drug-based traditional protocol still plays an important role clinically. Therefore, combining these two different kinds of treatments to achieve the maximal benefits to cancer patients has been increasingly discussed [34, 35], which would guide better designs for future synergistic cancer therapies [36–38].

In the present work, we thus attempted to combine Vγ9Vδ2 T cells and cis-platinum (CDDP) to treat breast cancer MDA-MB-231 cells. Since CDDP (240 μM) had similar inhibition ability (~ 20%) to γδ T cell (E:T = 5:1) against MDA-MB-231 cells, it implicates with that either CDDP or Vγ9Vδ2 T cell alone is not optimal way for breast cancer therapy clinically. However, the combination of CDDP and Vγ9Vδ2 T cells could more significantly suppress breast cancer cell growth (~ 40% of inhibition rate), showed the synergetic effects [36] of CDDP with Vγ9Vδ2 T cell on inhibiting breast cancer cell proliferation. These results could be implicated with two interpretations, one is that Vγ9Vδ2 T cells could strength cis-Platinum inhibition activity against breast cancer cell MDA-MB-231; the other one is that CDDP pretreated MDA-MB-231 cells became more sensitive to Vγ9Vδ2 T cells.

One of the major difficulties for tumor treatment lies that cancer cells could produce large amount of reactive oxygen species (ROS) due to their rapid growth, which results from imbalanced redox homeostatic state (mild pro-oxidative state) [39–41]. It’s reported that mitochondria and reactive oxygen species (ROS) produced by mitochondria are closely correlated with oncogenesis and cancer malignancy [42–44], and the abundant ROS in cancer cells could suppress the immune responses of cytotoxic immune cells. Although previous work showed CD8^+^ T cells induced cancer cell death in a mitochondria independent manner [45], how Vγ9Vδ2 T cell affect mitochondrial function of cancer cell remains largely unclear. In this context, reduction in ROS production of MDA-MB-231 cells in this work could be implicated with impairment of mitochondrial function in the presence of CDDP or Vγ9Vδ2 T cell. Moreover, our research results evidenced that CDDP or Vγ9Vδ2 T cells could significantly reduce not only mitochondrial mass but also mitochondrial membrane potential of MDA-MB-231 cells. The loss of mitochondrial membrane potential leads to cell apoptosis and cell death eventually. This explained why treatments of MDA-MB-231 cells by CDDP, Vγ9Vδ2 T cells, or their combination led to decreases in ROS production. Nevertheless, our work exhibited that Vγ9Vδ2 T cell could further potentiate CDDP inhibition against breast cancer cell MDA-MB-231, and the induced impairments in mitochondrial function played crucial role in this process.

After investigations at the scale of large number of cells, further work from the single cell view would provide us new knowledge on understanding how Vγ9Vδ2 T cells potentiated CDDP inhibition against MDA-MB-231 cells. The practicable nanobiotechnology, atomic force microscopy, could present new angle at the single cell level to reveal the veil on how cell membrane surface nanostructures were altered in the context of treatments. In our results, we discovered that alterations of membrane nanostructures induced by CDDP or Vγ9Vδ2 T cells were not visually apparent, however, further statistical analyses based on average roughness revealed gradual loss of membrane intact nanostructures in the context of treatments. Furthermore, cytoskeletal destructions suggested that CDDP or Vγ9Vδ2 T cell treatment impaired cytoskeleton organization more significantly than membrane surface ultrastructure. These visualized observations from the single cell level implied that cytoskeleton would be more sensitive indicator than membrane ultrastructure for revealing growth inhibition of MDA-MB-231 cells in the context of anti-tumor drug or cytotoxic immune cells. Additionally, both ultrastructural and cytoskeletal visualizations suggested that Vγ9Vδ2 T cells could further strengthen cytotoxicity of CDDP against breast cancer cell MDA-MB-231, implicating with the great clinical value to supplement γδ T cells to the current chemotherapy protocols to benefit tumor patients with breast cancer eventually. Altogether, the present results implied that combination had significant superiority compared with CDDP or Vγ9Vδ2 T cell alone, which provided scientific clues for clinically testing the advantages of CDDP plus Vγ9Vδ2 T cell strategy in the future.

## Conclusion

The present work for the first time demonstrated an in vitro paradigm for combining cis-platinum and Vγ9Vδ2 T cells to treat breast cancer cells, which clearly showed that Vγ9Vδ2 T cells could greatly strengthen cisplatin inhibition activity against breast cancer MDA-MB-231 cells. Our results revealed that the growth inhibition of MDA-MB-231 cells involved in mitochondrial dysfunction, cell membrane ultrastructural destruction and cytoskeleton disorganization.

## Data Availability

We declared that materials described in the manuscript, including all relevant raw data, will be freely available to any scientist wishing to use them for noncommercial purposes, without breaching participant confidentiality. The datasets generated/analyzed during the current study are available.

## Abbreviations

CDDP: Cis-platinum
PBMCs: Peripheral blood monocyte cells
FBS: Fetal bovine serum
AFM: Atomic force microscopy
ROS: Reactive oxygen species

## References

[1] Shaked Y (2016). Balancing efficacy of and host immune responses to cancer therapy: the yin and yang effects. Nat Rev Clin Oncol.

[2] Tonnessen-Murray CA (2019). Chemotherapy-induced senescent cancer cells engulf other cells to enhance their survival. J Cell Biol.

[3] Rodier F (2009). Persistent DNA damage signalling triggers senescence-associated inflammatory cytokine secretion. Nat Cell Biol.

[4] Vinothini K (2019). Dual role of lanthanum oxide nanoparticles functionalized co-polymeric micelle for extended anti-cancer drug delivery. ChemistrySelect.

[5] Kannan K (2020). Facile fabrication of CuO nanoparticles via microwave-assisted method: photocatalytic, antimicrobial and anticancer enhancing performance. Int J Environ Anal Chem.

[6] Praphakar RA (2018). A pH-sensitive guar gum-grafted-lysine-beta-cyclodextrin drug carrier for the controlled release of 5-flourouracil into cancer cells. J Mater Chem B.

[7] Rajan M (2016). Poly-carboxylic acids functionalized chitosan nanocarriers for controlled and targeted anti-cancer drug delivery. Biomed Pharmacother.

[8] Prabakaran S (2019). Polymethyl methacrylate–ovalbumin @ graphene oxide drug carrier system for high anti-proliferative cancer drug delivery. Appl Nanosci.

[9] Jeyaraj M (2016). Surface functionalization of natural lignin isolated from Aloe barbadensis Miller biomass by atom transfer radical polymerization for enhanced anticancer efficacy. RSC Adv.

[10] Magee MS, Snook AE (2014). Challenges to chimeric antigen receptor (CAR)-T cell therapy for cancer. Discov Med.

[11] Brudno JN, Kochenderfer JN (2018). Chimeric antigen receptor T-cell therapies for lymphoma. Nat Rev Clin Oncol.

[12] Neelapu SS (2018). Chimeric antigen receptor T-cell therapy - assessment and management of toxicities. Nat Rev Clin Oncol.

[13] Sebestyen Z (2020). Translating gammadelta (gammadelta) T cells and their receptors into cancer cell therapies. Nat Rev Drug Discov.

[14] Park (2020). Genomic methods identify homologous recombination deficiency in pancreas adenocarcinoma and optimize treatment selection. Clin Cancer Res..

[15] Tran E, Longo DL, Urba WJ (2017). A Milestone for CAR T Cells. N Engl J Med.

[16] Xu Y (2020). Allogeneic Vgamma9Vdelta2 T-cell immunotherapy exhibits promising clinical safety and prolongs the survival of patients with late-stage lung or liver cancer. Cell Mol Immunol.

[17] Alnaggar M (2019). Allogenic Vgamma9Vdelta2 T cell as new potential immunotherapy drug for solid tumor: a case study for cholangiocarcinoma. J Immunother Cancer.

[18] Xiang Z, Tu W (2017). Dual face of Vgamma9Vdelta2-T cells in tumor immunology: anti- versus pro-tumoral activities. Front Immunol.

[19] Fisher JP (2014). gammadelta T cells for cancer immunotherapy: a systematic review of clinical trials. Oncoimmunology.

[20] Silva-Santos B, Mensurado S, Coffelt SB (2019). gammadelta T cells: pleiotropic immune effectors with therapeutic potential in cancer. Nat Rev Cancer.

[21] Gogoi D, Chiplunkar SV (2013). Targeting gamma delta T cells for cancer immunotherapy: bench to bedside. Indian J Med Res.

[22] Aggarwal R (2013). Human Vgamma2Vdelta2 T cells limit breast cancer growth by modulating cell survival-, apoptosis-related molecules and microenvironment in tumors. Int J Cancer.

[23] Benzaid I (2011). High phosphoantigen levels in bisphosphonate-treated human breast tumors promote Vgamma9Vdelta2 T-cell chemotaxis and cytotoxicity in vivo. Cancer Res.

[24] Meraviglia S (2010). In vivo manipulation of Vgamma9Vdelta2 T cells with zoledronate and low-dose interleukin-2 for immunotherapy of advanced breast cancer patients. Clin Exp Immunol.

[25] Mattarollo SR (2007). Chemotherapy and zoledronate sensitize solid tumour cells to Vgamma9Vdelta2 T cell cytotoxicity. Cancer Immunol Immunother.

[26] Hu Y (2019). Selenium nanoparticles as new strategy to potentiate gammadelta T cell anti-tumor cytotoxicity through upregulation of tubulin-alpha acetylation. Biomaterials.

[27] Kouakanou L (2019). Vitamin C promotes the proliferation and effector functions of human gammadelta T cells. Cell Mol Immunol.

[28] Wu Y, Sims RC, Zhou A (2014). AFM resolves effects of ethambutol on nanomechanics and nanostructures of single dividing mycobacteria in real-time. Phys Chem Chem Phys.

[29] Wu YZ (2008). The analysis of morphological distortion during AFM study of cells. Scanning.

[30] Wu YZ (2010). BRMS1 expression alters the ultrastructural, biomechanical and biochemical properties of MDA-MB-435 human breast carcinoma cells: An AFM and Raman microspectroscopy study. Cancer Lett.

[31] Wu YZ, Zhou AH (2009). In situ, real-time tracking of cell wall topography and nanomechanics of antimycobacterial drugs treated Mycobacterium JLS using atomic force microscopy. Chem Commun.

[32] .McEwen GD, et al. Subcellular spectroscopic markers, topography and nanomechanics of human lung cancer and breast cancer cells examined by combined confocal Raman microspectroscopy and atomic force microscopy. Analyst. 2013;138:787–797. 10.1039/c2an36359c 23187307

[33] Wu YZ (2009). Time-dependent surface adhesive force and morphology of RBC measured by AFM. Micron.

[34] Gotwals P (2017). Prospects for combining targeted and conventional cancer therapy with immunotherapy. Nat Rev Cancer.

[35] Ott PA (2017). Combination immunotherapy: a road map. J Immunother Cancer.

[36] Emens LA, Middleton G (2015). The interplay of immunotherapy and chemotherapy: harnessing potential synergies. Cancer Immunol Res.

[37] Wu J, Waxman DJ (2018). Immunogenic chemotherapy: Dose and schedule dependence and combination with immunotherapy. Cancer Lett.

[38] Yu WD (2019). Mechanisms and therapeutic potentials of cancer immunotherapy in combination with radiotherapy and/or chemotherapy. Cancer Lett.

[39] Vucetic M (2017). The central role of amino acids in cancer redox homeostasis: vulnerability points of the cancer redox code. Front Oncol.

[40] Santos AL, Sinha S, Lindner AB (2018). The good, the bad, and the ugly of ROS: new insights on aging and aging-related diseases from eukaryotic and prokaryotic model organisms. Oxid Med Cell Longev.

[41] Kumari S (2018). Reactive oxygen species: a key constituent in cancer survival. Biomark Insights.

[42] Momcilovic M (2019). In vivo imaging of mitochondrial membrane potential in non-small-cell lung cancer. Nature.

[43] Porporato PE (2018). Mitochondrial metabolism and cancer. Cell Res.

[44] Zhang BB (2015). Mitochondrial membrane potential and reactive oxygen species in cancer stem cells. Fam Cancer.

[45] Jaime-Sanchez P (2020). Cell death induced by cytotoxic CD8(+) T cells is immunogenic and primes caspase-3-dependent spread immunity against endogenous tumor antigens. J Immunother Cancer.

